# Drug repositioning using drug-disease vectors based on an integrated network

**DOI:** 10.1186/s12859-018-2490-x

**Published:** 2018-11-21

**Authors:** Taekeon Lee, Youngmi Yoon

**Affiliations:** Department of Computer Engineering, Gachon University, 5-22Ho, IT college, 1324 Seongnam-daero, Seongnam-si, 13120 South Korea

**Keywords:** Network biology, Drug repositioning, Gene regulation, Protein interaction

## Abstract

**Background:**

Diverse interactions occur between biomolecules, such as activation, inhibition, expression, or repression. However, previous network-based studies of drug repositioning have employed interaction on the binary protein-protein interaction (PPI) network without considering the characteristics of the interactions. Recently, some studies of drug repositioning using gene expression data found that associations between drug and disease genes are useful information for identifying novel drugs to treat diseases. However, the gene expression profiles for drugs and diseases are not always available. Although gene expression profiles of drugs and diseases are available, existing methods cannot use the drugs or diseases, when differentially expressed genes in the profiles are not included in their network.

**Results:**

We developed a novel method for identifying candidate indications of existing drugs considering types of interactions between biomolecules based on known drug-disease associations. To obtain associations between drug and disease genes, we constructed a directed network using protein interaction and gene regulation data obtained from various public databases providing diverse biological pathways. The network includes three types of edges depending on relationships between biomolecules. To quantify the association between a target gene and a disease gene, we explored the shortest paths from the target gene to the disease gene and calculated the types and weights of the shortest paths. For each drug-disease pair, we built a vector consisting of values for each disease gene influenced by the drug. Using the vectors and known drug-disease associations, we constructed classifiers to identify novel drugs for each disease.

**Conclusion:**

We propose a method for exploring candidate drugs of diseases using associations between drugs and disease genes derived from a directed gene network instead of gene regulation data obtained from gene expression profiles. Compared to existing methods that require information on gene relationships and gene expression data, our method can be applied to a greater number of drugs and diseases. Furthermore, to validate our predictions, we compared the predictions with drug-disease pairs in clinical trials using the hypergeometric test, which showed significant results. Our method also showed better performance compared to existing methods for the area under the receiver operating characteristic curve (AUC).

**Electronic supplementary material:**

The online version of this article (10.1186/s12859-018-2490-x) contains supplementary material, which is available to authorized users.

## Background

The development of new drugs is a very lengthy and costly process and the average number of new drugs approved by the US Food and Drug Administration (FDA) per year has declined since the 1990s [1]. Computational biology studies have been progressed to reduce the cost and time of traditional drug development.

Drug repositioning, defined as identifying novel indications for existing drugs, is a good alternative for overcoming the limitations of traditional methods. A variety of approaches are used in computational drug repositioning studies. Network-based analysis is the most widely applied strategy [2]. Diverse properties of drugs and diseases have been used to construct networks for drug repositioning. Several previous studies employed drug network and/or disease network based on side effects, phenotypes or pathways, [3–6]. Their networks are not able to include drugs of which those properties are not known such as clinical compound and failed drugs in clinical trials. There are also other network-based studies using biomolecular networks [7–9]. These studies utilized relationships between drugs and diseases on the biomolecule networks. However, most existing approaches using the biomolecular network did not consider the characteristics of interactions between genes, such as activation, inhibition, or binding. They calculated similarities between drugs (or diseases) using the distance of shortest path from a drug to a disease on a network and then identify candidate drugs for diseases using the guilt-by-association method based on the similarities. These studies have proposed effective methods that can be applied to various drugs and diseases. However, considering interaction types between genes is important for discovering therapeutic associations of drug-disease pairs. Several studies used gene regulation information derived from gene expression profiles and obtained promising results for drug repositioning. Shigemizu et al. and Sirota et al. hypothesized that a drug is a potential treatment option for a disease if common genes are regulated oppositely by the drug and disease [10, 11]. They obtained gene expression data for drugs and diseases, and split the genes into up- and down-regulated classes based on expression differentials between normal and control tissue for each drug and disease. They next identified candidate drugs for various diseases. Under the same assumptions, Yu et al. determined the effects of drugs on each disease gene by exploring their networks rather than using gene expression data for the drugs [12]. Unlike previous network-based studies which did not consider the types of interaction between genes, they constructed a directed network with “activation” and “inhibition” edges for gene relationships. They considered not only the distances between the drug and disease by finding the shortest paths from the drug to each disease gene in the network, but also the types of shortest paths based on the relationships between genes. Their method showed good performance, but still required gene expression profiles for diseases. Additionally, although gene expression profiles for diseases are available, if their network does not contain differentially expressed genes, the diseases are not included in the method.

We proposed a novel method for identifying candidate drugs for diseases based on known disease-drug associations. Many biological network-based approaches have used diverse networks including PPI network, KEGG, gene co-expression network and integrated network [13, 14]. In this study, we used an integrated network consisting of protein interactions and gene regulations. The gene regulation and protein interaction data were collected from public databases providing diverse biological pathways. We converted heterogeneous IDs of genes and proteins in various databases to Entrez Gene ID and constructed a directed network with three types of edges defined as “positive”, “negative”, and “neutral” based on the relationships between molecules [15]. For drugs having a therapeutic association with a disease, we found the shortest paths from the drug to the disease genes in the network and calculated the weight and type of each shortest path. We constructed vectors representing how each disease gene was influenced by the drug for all drug-disease pairs. Next, we prepared a training set using the vectors to generate classifiers for identifying substitute drugs for known drugs. Our method conducted drug repositioning considering the relationship types between genes based on known drug-disease associations regardless of whether there are gene expression profiles for the drugs and disease. Thus, our method could be applied to a greater number of drugs and diseases compared to existing methods. Additionally, our predictions showed significant results in clinical trials enrichment tests [16]. Our model showed better areas under the receiver operating characteristic curve (AUCs) than existing studies for most diseases compared.

## Results

### A directed gene network

**Fig. 1:**
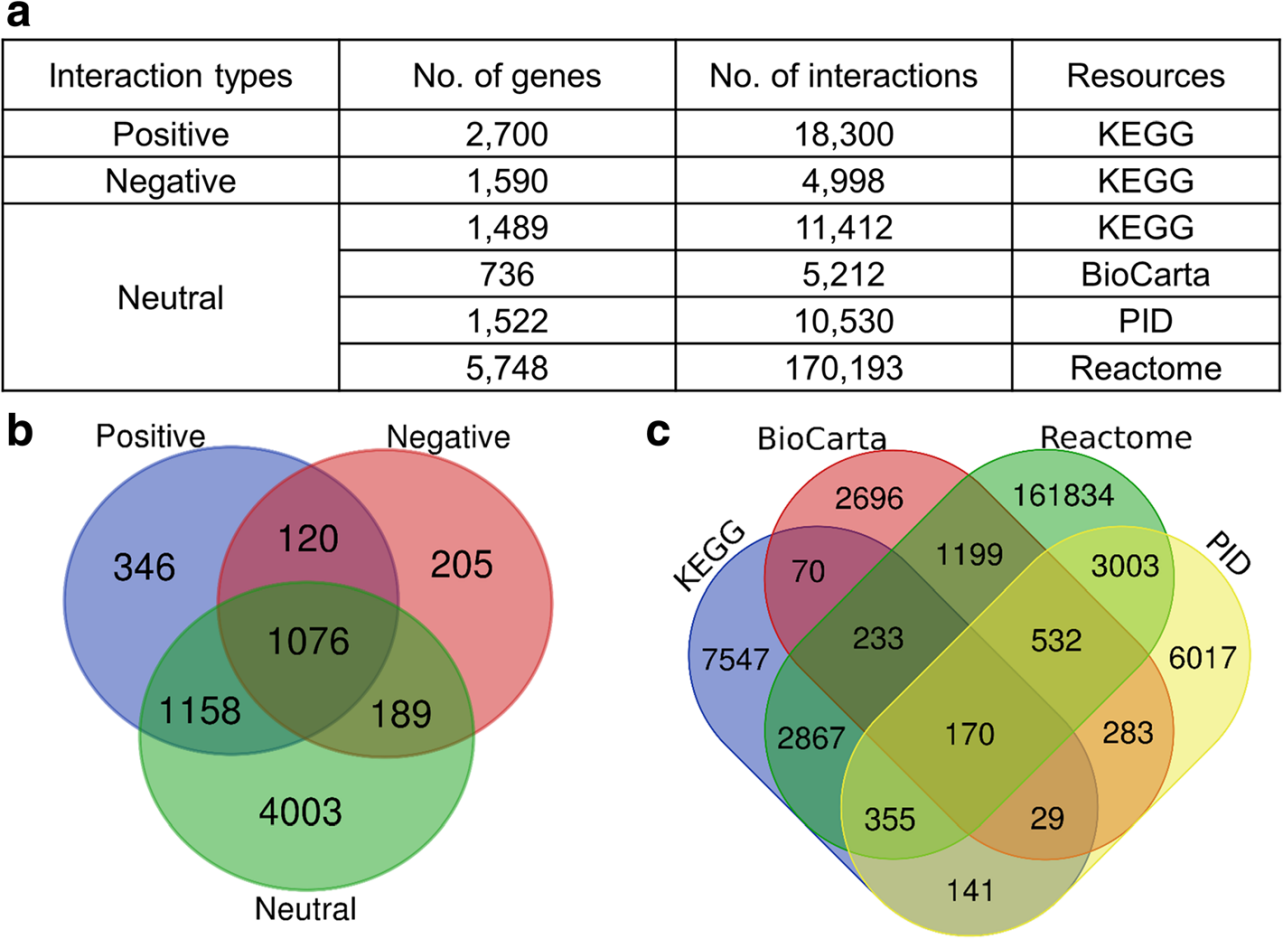
Summary of data used for our network (**a**) summary of data used for constructing a network. **b** Venn diagram representing the number of genes for each of three interaction types. **c** Venn diagram representing the number of “neutral” interactions

We constructed a directed network using interactions obtained from BioCarta [17], Reactome [18], the Pathway Interaction Database (PID) [19], and KEGG [13]. The genes and interactions used for constructing the network were summarized in Fig. 1. Existing studies considered “positive” and “negative” relationships between genes. However, the number of genes with “positive” or “negative” relationships was much smaller than the total number of genes and they did not sufficiently cover the full gene network. We defined binding interactions as “neutral” relationship, where binding interaction is not classified into control or conversion process by the databases. We integrated “neutral” interactions from four different databases (Fig. 1.a). The number of genes in the databases was 9350. Among them, only 3094 genes had interactions corresponding to “positive” or “negative” relationships. In contrast, our network covered 7097 genes by considering the three types of interactions (Fig. 1.b). The number of interactions corresponding to “neutral” was 186,976 (Fig. 1.c).

### Performance evaluation

We constructed classifiers for 298 diseases using the random forest, and for 296 diseases using the neural network. To evaluate performance of our model, we repeated 10-fold cross-validations 100 times for each disease and calculated the mean AUC of a disease. Table 1 represents the number of diseases that satisfies the given AUC range.

**Table 1:** The number of diseases that satisfies the given AUC range

Mean AUC	Random forest	Neural network
≥ 0.9	5	0
≥ 0.8	49	12
≥ 0.7	160	77
≥ 0.6	266	208
≥ 0.5	292	289
Total No. of diseases	298	296

The number of diseases showing AUC values above 0.6 was 266 of 298 diseases (89.3%) and 208 of 296 diseases (70.2%) for the random forest and neural network, respectively. Our method could be applied to any disease if the disease has known drugs. Particularly, we obtained AUC values greater than 0.9 for B-cell chronic lymphocytic leukemia, dysmenorrhea, urticarial, and psoriasis by random forest model. The mean AUC and standard deviation for each disease are shown in the Additional file 1.

### Prediction of candidate drugs for diseases and validation

In our method, random forest showed better performance than neural network. Thus, we used the classifiers learned by random forest algorithm to identify novel drugs for diseases.

For each disease, 100 classifiers were learned by random forest model using 100 different training sets, and AUCs of the classifiers were calculated using 10-fold cross-validation. We selected a classifier showing the best AUC and identified candidate drug-disease pairs using the classifier. If multiple classifiers showed same top AUC, we included all drug-disease pairs predicted by each of the best classifiers.

We sorted drug-disease pairs in descending order based on the probability that the pair was predicted to be “TRUE” by the classifier. If a drug-disease pair was predicted from multiple classifiers, we assigned the mean probability to the pair. Table 2 shows predicted drugs having high probabilities for five diseases of which mean AUC ≥ 0.9. The list of all of our candidate drug-disease pairs is provided by Additional file 2.

**Table 2:** Candidate drugs with high probabilities for diseases for which the mean AUC ≥ 0.9

Disease (MeSH)	Drug
Osteoarthritis (D010003)	Meclofenamic acid, Dihomo-gamma-linolenic acid, Diethylcarbamazine, Hyperforin, Niflumic Acid, Morphine, Codeine, Hydromorphone, Oxycodone, Fentanyl, Levorphanol, Remifentanil, 3-Methylthiofentanyl, Heroin, Carfentanil, 3-Methylfentanyl, Rizatriptan, Pramlintide, Sulfasalazine, Bimatoprost
Chronic lymphocytic leukemia (D015451)	Bleomycin, Olaparib, Nelarabine, Palbociclib, Zidovudine, Vorinostat, Azacitidine, Decitabine, Romidepsin, Lucanthone, SU9516, Panobinostat, Alclometasone, Fluorometholone, Rimexolone
Dysmenorrhea (D004412)	Amantadine, Memantine, Menadione, Mesalazine, Levallorphan, Butorphanol, Dextropropoxyphene, Sulfasalazine, Alfentanil, Progabide, Anileridine, Meclofenamic acid, Acetylsalicylic acid, Balsalazide, Vigabatrin, Levomethadyl acetate, Methadyl acetate, Ethylmorphine, Tapentadol, Asfotase Alfa
Psoriasis (D011565)	Dexamethasone, Fludrocortisone, Diethylstilbestrol, Danazol, Megestrol acetate, Prasterone, Fluticasone propionate, Raloxifene, Estradiol, Estriol, Estrone sulfate, Etonogestrel, Desogestrel, Medroxyprogesterone acetate, Ethynodiol diacetate, Norgestimate, Allylestrenol, Progesterone, Romidepsin, Vorinostat
Urticaria (D014581)	Acetazolamide, Esmolol, Atenolol, Methylergometrine, Practolol, Tetracosactide, Aprepitant, Enprofylline, Netupitant, Cetrorelix, Abarelix, Degarelix, Ganirelix, Dofetilide, Icatibant, Pimagedine, Belimumab, Zidovudine

To verify that our predictions are in accordance with current experimental knowledge, we enriched our candidate drug-disease pairs to drug-disease pairs in clinical trials using the hypergeometric test. Clinical trials data were obtained from the clinical trials web site (https://clinicaltrials.gov/) in XML format [16]. Our predictions significantly overlapped with those of clinical trials (p-val: 1.14E-08). In addition to clinical trials, we counted co-occurrence of our candidate drug-disease pairs from PubMed, an online database of citations for biomedical literature, using the easyPubMed package in R [20]. As a result, 23,249 out of 52,926 pairs overlapped with PubMed. For all candidate drug-disease pairs, PubMed counts and inclusion status were provided in clinical trials in Additional file 2.

**Fig. 2:**
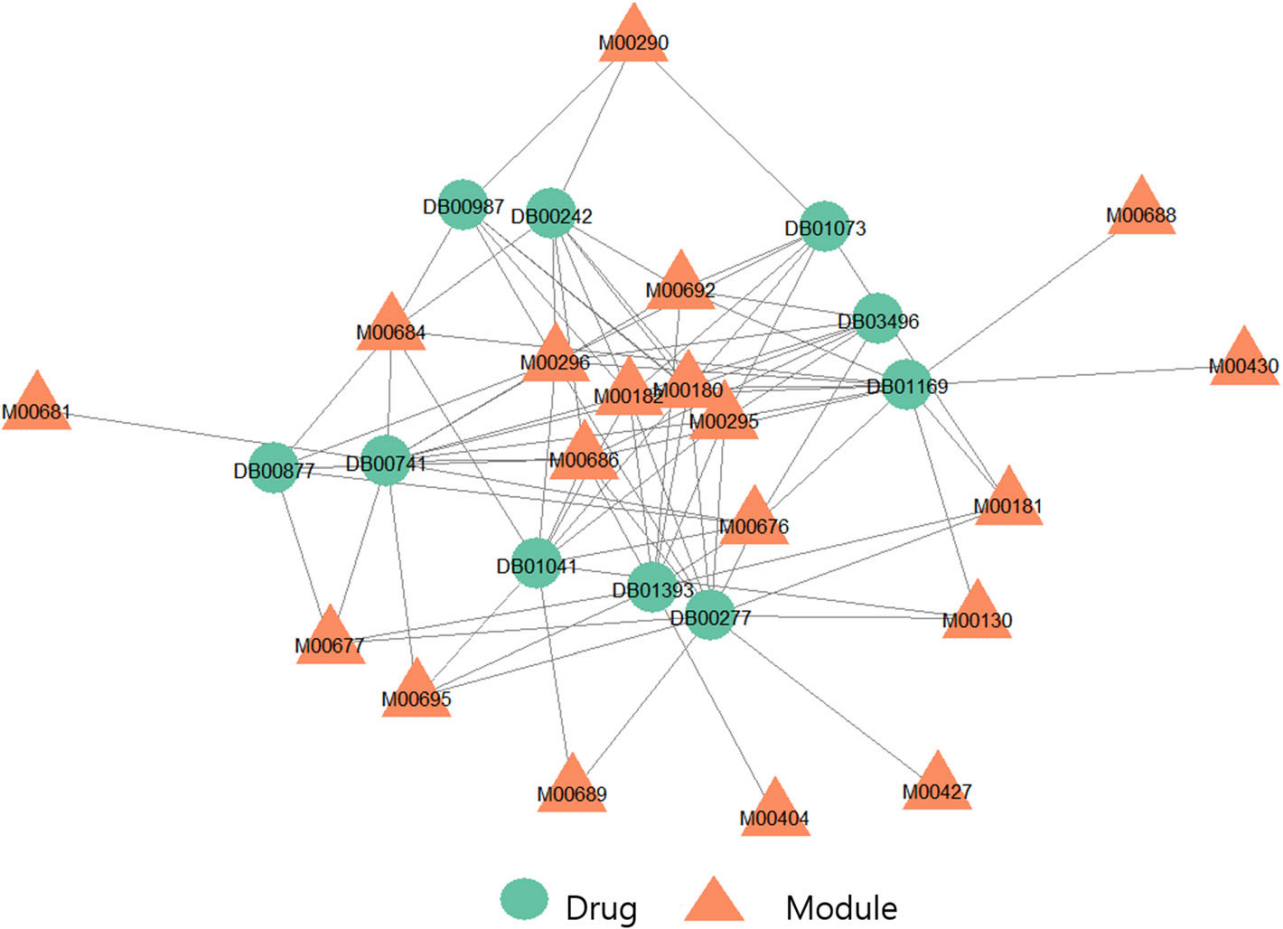
A network consisting of known drugs for B-CLL and enriched modules of the known drugs

Several studies tried to establish mechanisms of drugs for diseases using pathways between the drugs and diseases [21–23]. We showed that our candidate drugs were more functionally similar to known drugs for diseases than non-candidate drugs, using functional modules obtained from pathways between drugs and diseases. We obtained shortest pathways between drugs and diseases by exploring a gene network. For each drug-disease pair, we extracted significantly enriched modules from KEGG Module using gene lists of the pathway. For each disease, we compared candidate drugs and non-candidate drugs for functional similarity to known drugs using the Wilcoxon rank-sum test. As a result, our set of candidate drugs showed significantly higher similarity than the set of non-candidate drugs in 285 of 298 diseases (p-val < 0.05). Furthermore, the enriched modules of known and candidate drugs for a disease could be used for basis to support our candidate drug-disease pairs. As an example, we presented SU9516 (DB03428) for B-cell chronic lymphocytic leukemia (B-CLL, D015451). We used 10 known drugs for B-CLL and 19 modules was enriched more than once between the known drugs and the disease. We display the drugs and the modules as a network in Fig. 2. We considered the commonly enriched modules to play important roles in treating B-CLL. The average of modules degree was 4.63 and the Table 3 shows the name of 8 modules having a degree greater than the average, and their degree.

**Table 3:** Commonly enriched modules of known drugs for B-CLL

Module ID	Module name	Number of Degree
M00296	BER complex	9
M00180	RNA polymerase II, eukaryotes	9
M00182	RNA polymerase I, eukaryotes	9
M00295	BRCA1-associated genome surveillance complex (BASC)	9
M00686	Toll-like receptor signaling	8
M00676	PI3K-Akt signaling	7
M00692	Cell cycle - G1/S transition	6
M00684	JAK-STAT signaling	6

In our predictions, SU9516 (DB03428) for B-CLL had a high probability for treating B-CLL, but the pair was not found in clinical trials and PubMed. SU9516 is a specific inhibitor of cyclin-dependent kinases (CDK) including CDK1, CDK2, and CDK5. There were 9 enriched modules of SU9516 for B-CLL, and 7 out of the 9 modules overlapped with the 8 modules in Table 3. In other words, SU9516 is functionally similar to known drugs. Additionally, we found a case from literature that another CDK inhibitor, flavopiridol, had clinical activity for chronic lymphocytic leukemia, although the drug development was consequently discontinued in 2012 [24, 25]. Significantly enriched modules of known and candidate drugs with KEGG Module for 298 disease are available from (http://databio.gachon.ac.kr/tools/Datasets/). A list of enriched modules is available for each disease.

### Comparison with existing methods

**Fig. 3:**
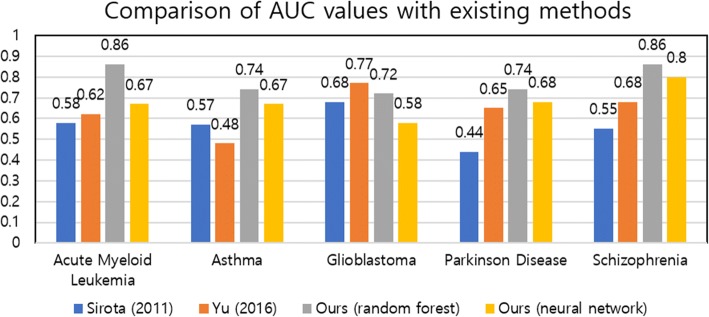
Comparison with existing methods

We compared our method with existing methods that considered gene regulation. Yu et al. [12] described the AUCs of diseases using their method and AUCs of the diseases using Sirota’s method [11]. Their method relied on gene expression data. Additionally, although gene expression profiles of diseases are available, the method could not use the diseases when differentially expressed genes in the profiles are not included in their network. Whereas, since we used 10-fold cross-validation, our method required that a disease should have at least 10 known drug-disease associations. Figure 3 represents AUCs of five common diseases used in their method and ours. For five common diseases, the number of disease genes and known drugs for each disease are provided in Table 4.

**Table 4:** The number of known drugs and disease gene for five common diseases

Disease	MeSH ID	The number of known drugs	The number of disease genes
Acute Myeloid Leukemia	D015470	28	76
Asthma	D001249	46	71
Glioblastoma	D005909	15	49
Parkinson Disease	D010300	35	62
Schizophrenia	D012559	57	138

In the comparison, we used mean AUC of 10-fold cross-validations repeated 100 times for each disease. Our method showed better performance for four diseases except for glioblastoma.

**Fig. 4:**
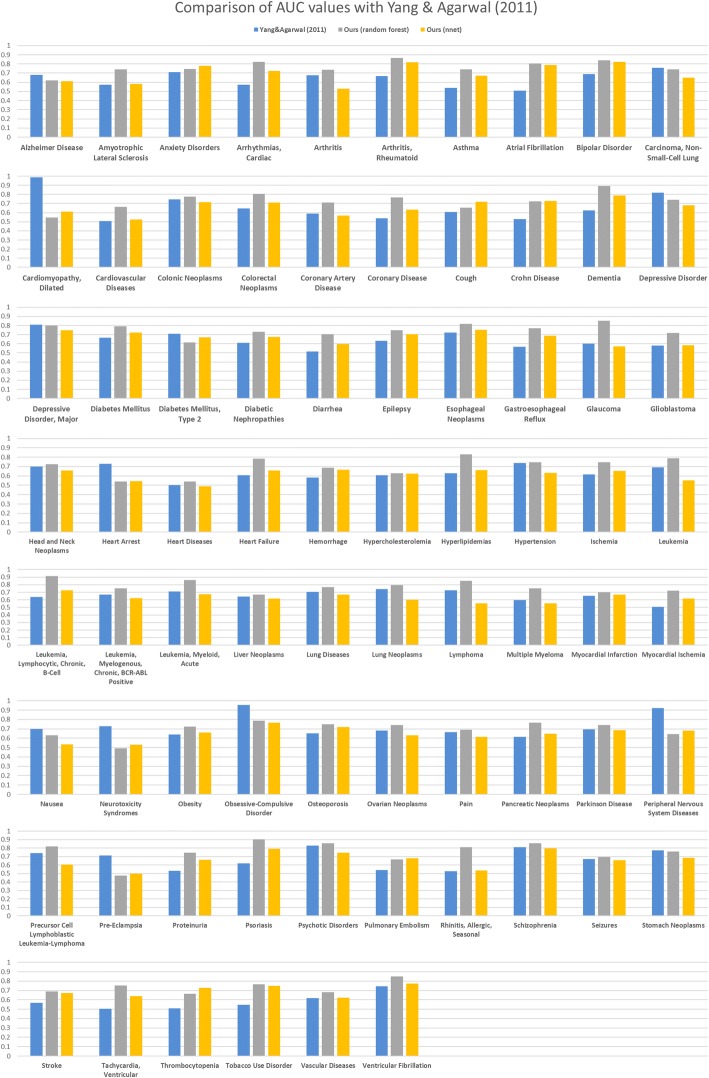
Comparison with Yang & Agarwal (2011)

Additionally, we compared our method with the drug repositioning model based on side effects. Yang and Agarwal [3] hypothesized that if the side effects associated with a drug D were also induced by a large number of drugs treating disease X, then drug D should be evaluated as a candidate for treating disease X. They demonstrated their method on drugs acquainted with side effects and then attempted to find indications of clinical candidates whose side effect information was unknown. They predicted side effects of the candidates based on compound structure and applied their method using the predicted side effects. Figure 4 represents AUCs of 76 common diseases of their method using predicted side effects and our method. Our method showed better performance for 63 out of 76 diseases (random forest) and 41 out of 76 diseases (neural network).

## Discussion

We required known drugs for diseases for learning of classifiers. We could not predict candidate drugs for diseases if they did not have known drugs enough to learn rules by machine learning methods. Even with this limitation, however, our method could be applied to a greater number of diseases than previous models considering drug-disease association using gene expression data since we did not use gene expression data. In addition to comparison with previous studies based on drug-disease genes relationships, we showed comparison with Yang and Agarwal based on side effects [3]. Although they partially overcame their limitation on drugs with no known side effects by using predicted side effects, our method showed better performance for many diseases.

Our method built classifiers to find candidate drugs for diseases using machine learning techniques. We used two machine learning algorithms, the random forest and the feed forward neural network. The predicting process of the classifiers was a ‘black box’. Therefore, it was hard to understand why the prediction was made by the classifiers [26]. However, explaining predictions is an important to trust and use the model [27]. Alternatively, we attempted to explain the rationale of predicted drug-disease pairs using functional modules from KEGG Module. We showed that our candidate drugs were more similar to known drugs compared with non-candidate drugs. As an example, we described that a predicted drug for B-CLL, SU9516, had most of commonly enriched modules of the known drugs for the disease.

Additionally, we found literature-based evidences for several predicted drug-disease pairs showing high probabilities among our predictions. We confirmed that the pairs were also promising candidates in studies without using computational methods.

Olaparib (DB05940) for B-CLL was found by our model. Olaparib which has been approved for treating ovarian cancer by the FDA, inhibits poly (ADP-ribose) polymerase (PARP). PARP is involved in DNA repair, cell death, chromatin functions and genomic stability [28]. PARP1 inhibitors selectivity kill cancer cells with defects in the homologous recombination repair pathway [29]. Weston et al. suggested that olaparib, a PARP inhibitor, is an appropriate agent for treating chronic lymphocytic leukemia [30].

Montelukast (DB00471) is used to treat seasonal allergic rhinitis (D006255). Allergic rhinitis is characterized by symptoms such as sneezing, itchy eyes, and watery rhinorrhea [31]. These symptoms negatively impact a person’s quality of life, although allergic rhinitis is not life-threatening. Cysteinyl-leukotrienes play an important role in allergic airway disease and montelukast is cysteinyl leukotriene type 1 receptor antagonist [32].

Initially developed as a treatment for asthma, montelukast has recently been used to treat allergic rhinitis [33]. Known drug-disease pairs used for seasonal allergic rhinitis did not include anti-leukotriene. However, anti-leukotriene was predicted to affect disease genes in a similar manner as anti-histamines in seasonal allergic rhinitis based on our method.

Meclofenamic acid (DB00939) for osteoarthritis (D010003) showed a high probability in our prediction. Osteoarthritis is the most common form of arthritis [34]. Meclofenamic acid is the most potent anti-inflammatory drug among the fenamic acids and belongs to a family of non-steroidal anti-inflammatory drugs. These drugs are recommended if symptoms are moderate to severe [35].

In future works, we plan to apply our method to predict unknown ADR (adverse drug reaction) of drugs. Predicting ADRs is considered as an important subject, because the unexpected effects of drugs can cause a severe problem to patients. In addition, ADRs of drugs are major causes of failure in drug development. ADRs are phenotypic responses of the human organism to drug treatment, in common with drug therapeutic indications. We can collect ADR-related genes, and known ADR-drug associations from public databases. Those data can be replaced with disease genes and known drug-disease associations used for drug repositioning.

## Conclusions

In this paper, we have presented a method using an integrated network for predicting novel indications of drugs. Our method contributes to drug repositioning for drugs which cannot be used in previous methods which required diverse properties of drugs, such as gene expression, indication, and side effects. We also considered the degree of genes and interaction types between genes unlike most existing methods using biomolecular networks which used only the distance between target genes and disease genes. Our method is able to differentiate between drugs having same targets but have different interaction types with the targets.

## Methods

### Datasets

Drug-target interactions were obtained from DrugBank [36] and the targets were mapped by their Entrez gene ID numbers [15]. Interactions between drugs and targets were assigned to the “positive” or “negative” as described by Torres et al. [37]. Additionally, we assigned “neutral” to interactions corresponding to binding. Unassigned interactions were not used in our method. Thus, 4248 interactions including 1163 drugs and 584 targets were used from among all drug-target interactions obtained from DrugBank.

Disease genes were obtained from curated gene-disease associations of DisGeNet [38]. Diseases were mapped by MeSH terms. We collected known drug-disease associations from chemical-disease associations of CTD [39]. Chemical were mapped by DrugBank ID. In this study, we used diseases with more than 10 known drugs only for 10-fold cross-validation. Briefly, we used 7083 associations between 1163 drugs and 298 diseases in our method.

### Constructing a directed network considering interaction type

We constructed a directed gene network by integrating genes and interactions between genes from BioCarta [17], Reactome [18], PID [19], and KEGG [13] using the Graphite tool [40], which was used for converting pathway topology to the gene network in R. The Graphite provided direction of interactions between genes, which were classified as one of two types: “directed” or “undirected”. In the case of “undirected”, if there was a relationship between gene A and gene B, we added both relationships from gene A to gene B and from gene B to gene A in our network. Each node represented a gene, and interactions between genes were expressed as edges. Edges consisted of three types, depending on relationships from a gene to another gene. As described by Yu et al., we considered a relationship as “positive” when activation or expression was recorded and as “negative” when inhibition or repression was recorded [12]. Additionally, we also considered relationships as “neutral” when they corresponded to binding. The binding interaction represented a binding between multiple elements, and was not classified into control or conversion process by the databases, unlike other interactions. There were differences in interaction terms between KGML and BioPax which are languages for representing biological pathways. We collected “positive” and “negative” edges from KEGG only and “neutral” edges from four databases mentioned above. Table 5 shows the interaction names from KEGG, and the conversion types in our network.

**Table 5:** Interaction names from KEGG and conversion types in our network

Interaction name	Interaction type in our network
Activation	Positive
Expression	Positive
Repression	Negative
Inhibition	Negative
Binding	Neutral
Phosphorylation	–
Indirect effect	–
Dissociation	–
Dephosphorylation	–
Ubiquitination	–
Missing interaction	–
Methylation	–
Glycosylation	–
State change	–

### Finding shortest paths from a target gene to a disease gene

To calculate the associations between a drug and a disease, we found the shortest paths from target genes to disease genes in the directed network. We presumed that neutral relationships maintain general actions in the body and that drugs affect diseases by regulating disease-related genes through activation or inhibition. Thus, we constrained the shortest paths between the drug and disease genes from connecting without at least one “positive” or “negative” edge.

We found the shortest paths in different manners according to the following two cases:

1. When a drug-target interaction was neutral, we added a constraint in which at least one positive or negative edge between the target gene and disease gene was required to prevent a path from the target to the disease gene to be neutral.
2. When the drug-target interaction was not neutral, we removed the constraint so that paths consisting of only neutral edges are allowed.

**Fig. 5:**
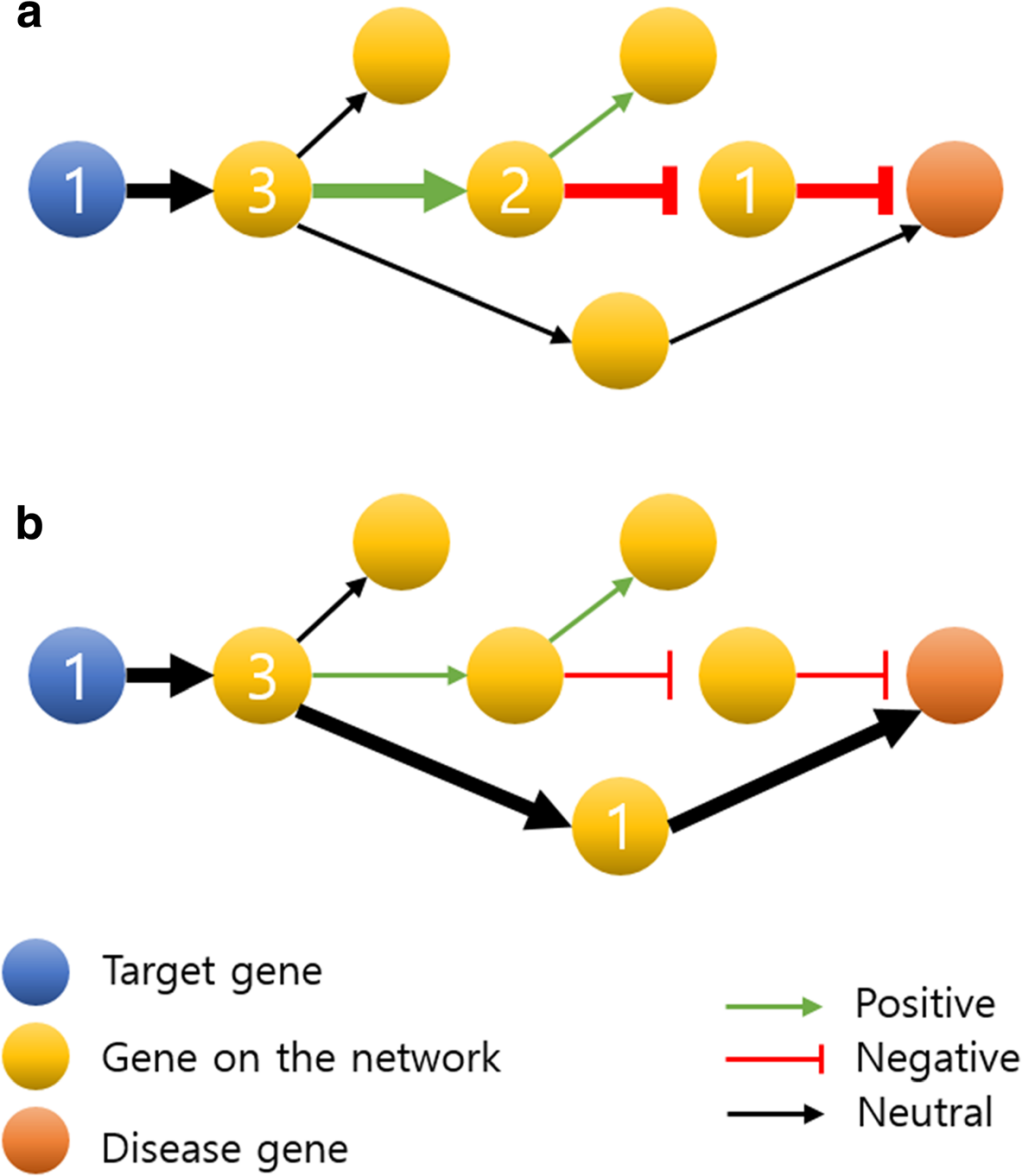
Finding shortest path from a target gene to a disease gene Edges of the shortest path are shown in bold. Out-degree of a node on the shortest path is denoted. a is a case in which the drug-target interaction is neutral. In this case, we required a path to have at least one positive or negative edge. **b** shows other cases in which a drug-target interaction is not neutral. In this case, the shortest path consisting of only neutral edges was allowed

Figure 5 represents the processes of finding shortest path in two cases of drug-target interactions. To quantify the associations between a target gene and a disease gene, we calculated the types and weights of the shortest paths, respectively. The type of a path was calculated by only “positive” and “negative” relationships between genes comprising the path. Thus, we assigned 1 to each positive edge and − 1 to each negative edge in the path. We defined the path type by multiplying the values corresponding to edges on the path. Namely, T denoted a type of path and E was a set of non-neutral edges in the path. T was determined using the following eq. (1).

1 $$ \mathrm{T}={\prod}_{i=1}^n{e}_i\left(\forall {e}_i\in E,\mathrm{E}\ne \varnothing \right) $$

We used the product of the reciprocals of out-degree of the corresponding node on a path as the path weight. The out-degree of a node is the number of interaction from the node to adjacent nodes. W denoted the path weight and *d*_*i*_ was the out-degree of the *i*_*th*_ node in the path composed of n nodes. W was calculated using eq. (2) as follows:

2 $$ \mathrm{W}={\prod}_{i=1}^n\frac{1}{d_i} $$

To consider the effects of all shortest paths from a target gene to a disease gene, we summarized the weights of each path considering the type of the path when there were multiple paths between the target gene and disease gene. V indicated the extent to which a disease gene was affected by a target gene, while m denoted the number of paths from the target gene to the disease gene. V was calculated using eq. (3) as follows:

3 $$ \mathrm{V}={\sum}_{i=1}^m\left({T}_i\times {W}_i\right) $$

### Building a vector for each drug-disease pair

**Fig. 6:**
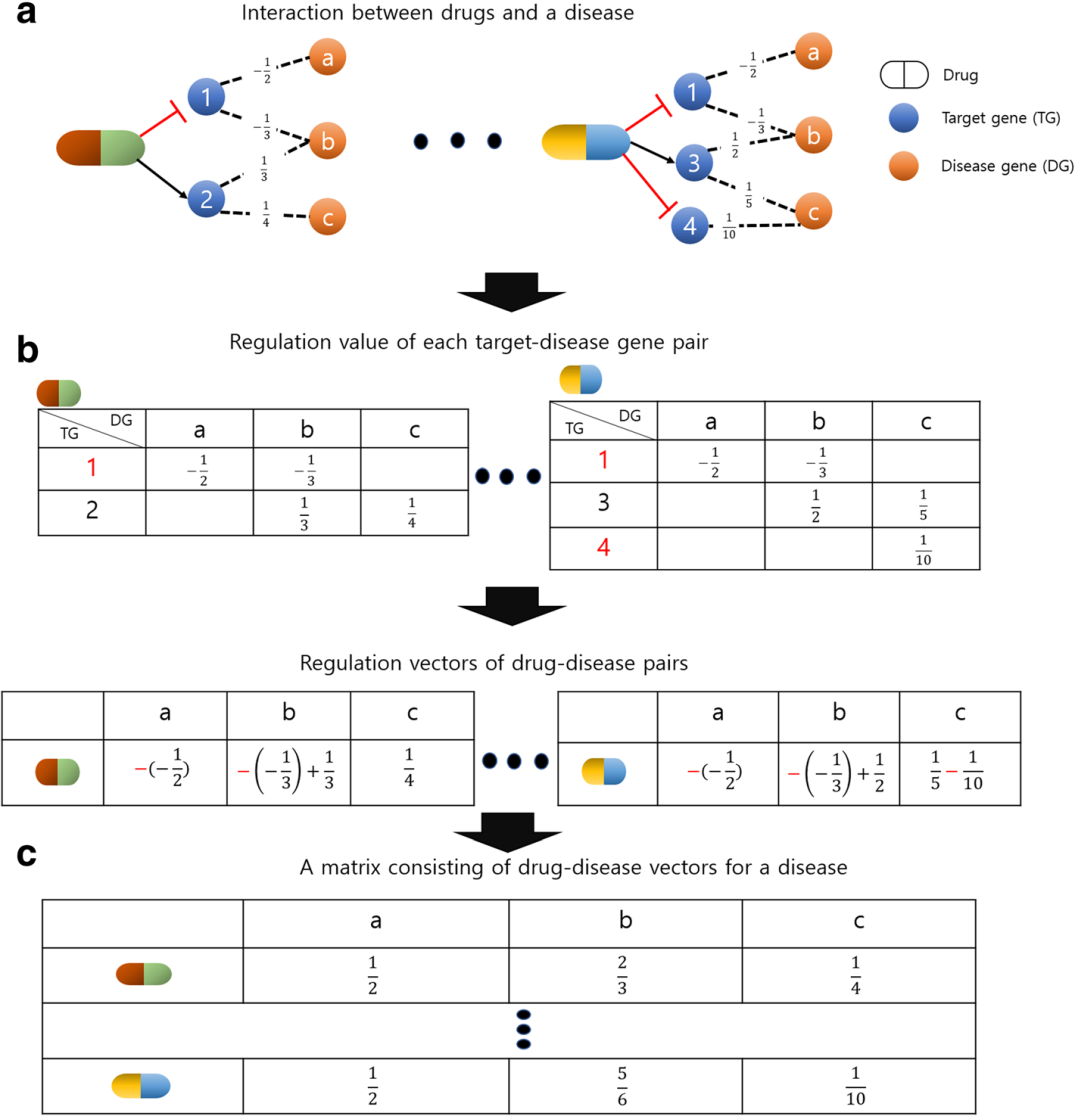
Outline flow chart for building a vector of a drug-disease pair.**a** represents interactions between drugs and a disease. We determined the type and weight for each target gene-disease gene pair by finding the shortest path from a target gene to a disease gene. **b** shows the process of building regulation vector using regulation values and interaction types for target gene-disease gene pairs. The value for each target gene-disease gene pair was multiplied by − 1 when the type of interaction between a drug and the target gene was inhibition. **c** shows a matrix consisting of drug-disease vectors for a disease. The size of each vector for a disease was determined by the number of its disease genes, and a group of vectors could be expressed as a matrix

To express the effects of a drug on a disease, we built a vector for each drug-disease pair (Fig. 6). The vector was composed of values in which disease genes were influenced by the drug. The values of the vector were calculated using V obtained from previous step. We multiplied the V by 1 for positive and − 1 for negative drug-target interactions. If a drug-target interaction was neutral, we did not perform the operation. When a drug had more than one target gene, we added the values by all target genes.

Namely, DV denoted a value reflecting the extent to which a disease gene was influenced by a drug, n denoted the number of target genes, and *sign*(*T*_*i*_) was value corresponding to the drug-*i*_*th*_ target interaction. DV was calculated as follows:

4 $$ DV={\sum}_{i=1}^n\mathit{\operatorname{sign}}\left({T}_i\right)\bullet {V}_i $$

### Predicting candidate drugs and validation

To construct classifiers to identify novel drugs for diseases using machine learning, we prepared training sets for each disease based on drug vectors. We assigned “TRUE” to a class for vectors corresponding to drugs in known drug-disease associations as positive set. We extracted drugs not included in the positive set. The number of drugs in this set was 3-fold larger than that in the positive set. We assigned “FALSE” to the class for the extracted drugs as the negative set. Next, we made a training set using positive set and negative set for constructing a classifier. To preprocess the training set, if a specific disease gene was not associated with any drug in the training set, we excluded the column corresponding to the disease gene. We applied the random forest [41] and neural network models [42] to the training set using the caret package [43] in R. The Caret package itself tunes parameters of diverse models for optimal results. The random forest is a supervised learning algorithm and can be used for both classification and regression. For random forest, we used *mtry* tuned by Caret. *mtry* is the number of variables randomly sampled as candidates at each split, and it is different in value depending on a disease and training sets for 10-fold cross-validation. We used feed-forward neural networks with a single hidden layer and the number of hidden unit and weight decay tuned by Caret. We performed 10-fold cross-validation for each model, and classifiers showing a higher AUC were selected for identification of novel drugs for each disease. To obtain robust results compared to existing models, this process was repeated 100 times. We used the mean AUC of repeated processes for comparison with existing disease. To demonstrate that our predicted drug-disease pairs were significant, we compared our prediction with drug-disease pairs in clinical trials using the hypergeometric test. Among our predicted pairs, we only used drugs and diseases included in clinical trials.

**Fig. 7:**
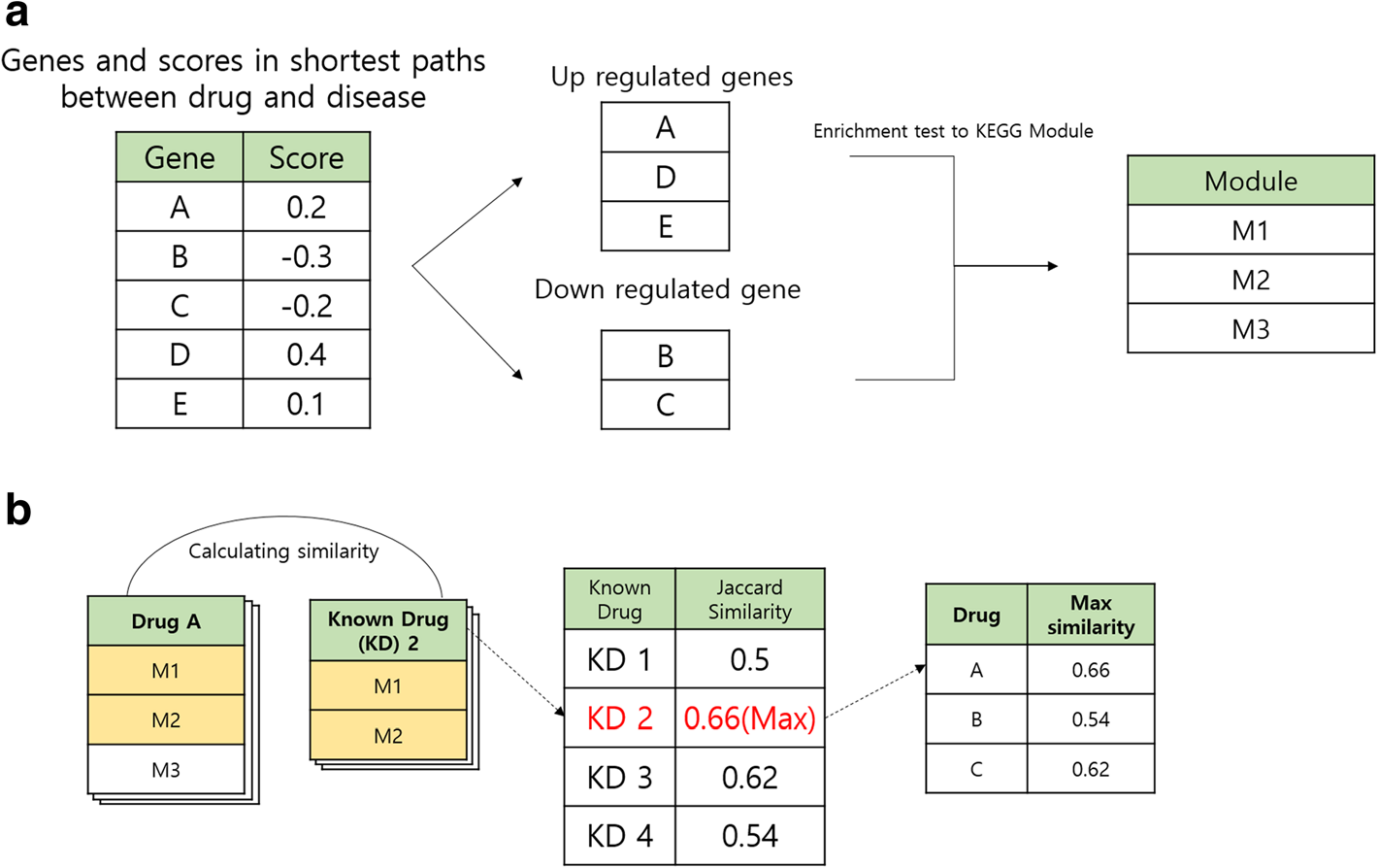
Data preprocessing for Wilcoxon rank-sum test. **a**) Extraction of significant modules for drug-disease pairs **b**) Assigning the highest from similarity scores with known drugs to each drug

Additionally, in order to show the significance of our prediction, we compared functional similarities of candidate drugs and known drugs with functional similarities of non-candidates and known drugs for all 298 diseases, respectively. We extracted statistically significant modules based on the enrichment test of genes in the shortest paths between a drug and a disease to KEGG Module, which is a collection of manually defined functional units. Hong et al. showed that analyzing up- and down-regulated genes separately was more powerful for detecting significant pathways than analyzing all genes [44]. We also performed separately enrichment test of up- and down-regulated genes to KEGG Module (Fig. 7.a) [13]. We calculated Jaccard similarities between candidate drugs and each known drug based on the obtained modules. Next, the candidate drugs were assigned the highest value among similarities with known drugs (Fig. 7.b). The same process shown in Fig. 7 was applied for non-candidate drugs. To show that candidate drugs are more functionally similar to known drugs than non-candidate drugs, we used the Wilcoxon rank-sum test, which is widely used to compare two groups (one-tailed, *p*-value < 0.05).

## Additional files

Additional file 1: The mean AUC and standard deviation from repeating 10-fold cross-validations for each disease using random forest and neural network. (XLSX 31 kb)

Additional file 2: Predicted drugs and probabilities corresponding to the drugs for diseases. (XLSX 2039 kb)

## Abbreviations

AUC: Area under the receiver operating characteristic curve
B-CLL: B-Cell chronic lymphocytic leukemia
DG: Disease gene
KD: Known drug of disease
PID: The pathway interaction database
TG: Target gene
